# Assessing function in patients undergoing joint replacement: a study protocol for a cohort study

**DOI:** 10.1186/1471-2474-13-220

**Published:** 2012-11-13

**Authors:** Vikki Wylde, Ashley W Blom, Stijn Bolink, Luke Brunton, Paul Dieppe, Rachael Gooberman-Hill, Bernd Grimm, Cindy Mann, Erik Lenguerrand

**Affiliations:** 1 Musculoskeletal Research Unit, School of Clinical Sciences, University of Bristol, Avon Orthopaedic Centre, Southmead Hospital, Bristol BS10 5NB, UK; 2 AHORSE Foundation, Department Orthopaedics, Atrium Medical Center Heerlen, Heerlen, the Netherlands; 3 Peninsula Medical School, Universities of Exeter and Plymouth, Plymouth, PL6 8BU, UK; 4 Musculoskeletal Research Unit, North Bristol NHS Trust, Avon Orthopaedic Centre, Southmead Hospital, Bristol, BS10 5NB, UK

**Keywords:** Joint replacement, Hip, Knee, Disability, Pain, Function, Outcome, Self-report, Performance tests, Motion analysis

## Abstract

**Background:**

Joint replacement is an effective intervention for people with advanced arthritis, although there is an important minority of patients who do not improve post-operatively. There is a need for robust evidence on outcomes after surgery, but there are a number of measures that assess function after joint replacement, many of which lack any clear theoretical basis. The World Health Organisation has introduced the International Classification of Functioning, Disability and Health (ICF), which divides function into three separate domains: Impairment, activity limitations and participation restrictions. The aim of this study is to compare the properties and responsiveness of a selection of commonly used outcome tools that assess function, examine how well they relate to the ICF concepts, and to explore the changes in the measures over time.

**Methods/design:**

Two hundred and sixty three patients listed for lower limb joint replacement at an elective orthopaedic centre have been recruited into this study. Participants attend the hospital for a research appointment prior to surgery and then at 3-months and 1-year after surgery. At each assessment time, function is assessed using a range of measures. Self-report function is assessed using the WOMAC, Aberdeen Impairment, Activity Limitation and Participation Restriction Measure, SF-12 and Measure Yourself Medical Outcome Profile 2. Clinician-administered measures of function include the American Knee Society Score for knee patients and the Harris Hip Score for hip patients. Performance tests include the timed 20-metre walk, timed get up and go, sit-to-stand-to-sit, step tests and single stance balance test. During the performance tests, participants wear an inertial sensor and data from motion analysis are collected. Statistical analysis will include exploring the relationship between measures describing the same ICF concepts, assessing responsiveness, and studying changes in measures over time.

**Discussion:**

There are a range of tools that can be used to assess function before and after joint replacement, with little information about how these various measures compare in their properties and responsiveness. This study aims to provide this data on a selection of commonly used assessments of function, and explore how they relate to the ICF domains.

## Background

Lower limb total joint replacement (TJR) is one of the most commonly performed elective surgical procedures in the UK. In 2010, 76,759 hip replacements and 81,979 knee replacements were performed in the NHS [1], and it is predicted that due to increases in obesity and an aging community, these figures will rise considerably in the coming years [2]. TJR is an effective and cost-effective intervention for people with advanced arthritis [3,4], although recent evidence suggests that an important minority do not improve post-operatively [5]. This concern, along with the known variations in the provision of and outcomes from TJR [6,7] mean that there is an urgent need for robust evidence on outcomes after surgery to aid appropriate patient selection and to provide patients with realistic predictions of the likely results.

The outcome after a TJR can be assessed in a variety of different ways. In the early years of TJR common adverse events, such as infection, as well as prosthesis survival were the main issues of concern [8]. However, as prosthesis design and the control of adverse events improved, these issues become less important, and attention turned towards clinician administered tools, such as the Harris Hip Score [9] and American Knee Society Score [10], and more recently towards patient reported outcome measures (PROMs) [11]. But it is not clear what PROM domains should be assessed, or how we can best measure them.

TJR is generally undertaken to reduce pain and improve function for people with advanced osteoarthritis (OA) [12]. Pain and disability are inextricably linked in OA, as the pain is largely activity-related [13]. Although pain is often considered the most important aspect of outcome to assess, it is a purely subjective issue, and therefore difficult to ‘measure’. Function, on the other hand, is a more objective domain, and as it is closely related to pain, it would seem reasonable to concentrate our measurement efforts on function. However, there are many different ways of assessing function and a large number of variables which can influence it [14]. Furthermore, most of the standard ways used to assess function lack any clear theoretical basis [15], and there has been relatively little research on the relationships between the different approaches used.

**Figure 1:**
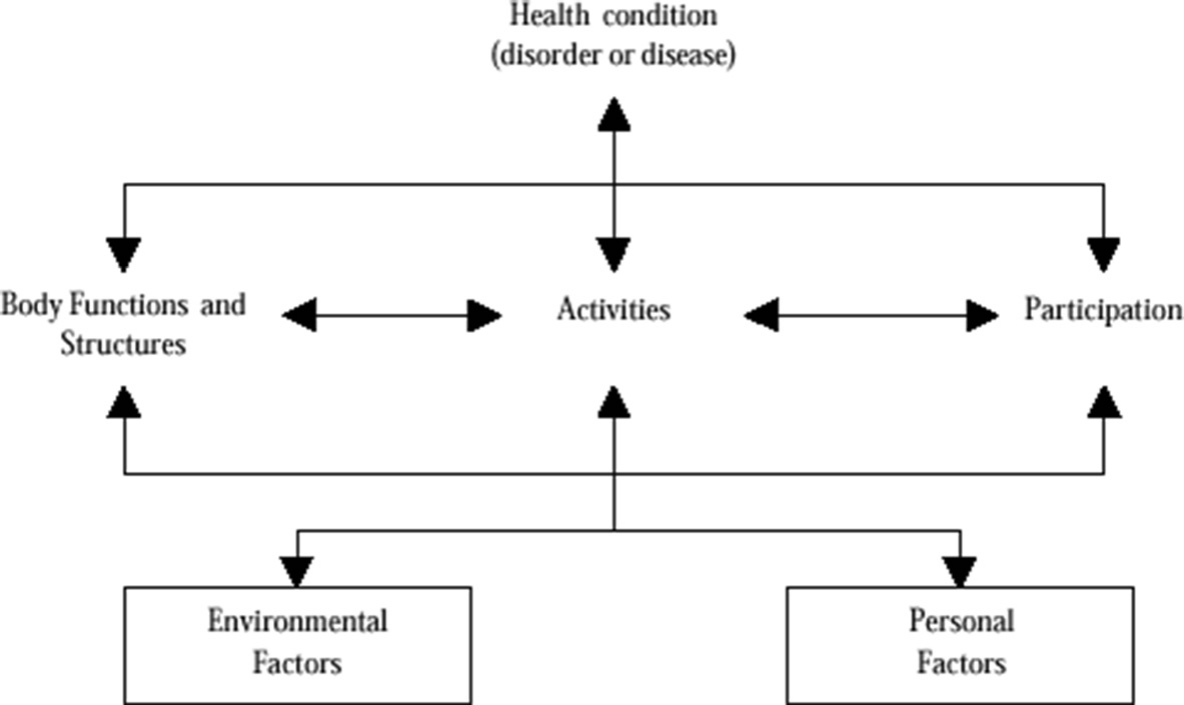
International Classification of Functioning, Disability and Health.

The World Health Organisation has introduced the International Classification of Functioning, Disability and Health (ICF) [16] which provides a theoretical framework on which to base the assessment of function. As shown in Figure 1, this framework splits function into three separate domains: impairment (I), activities limitations (A) and participation restrictions (P). The value of this in the context of TJR can be illustrated by taking the example of climbing a step, a common problem for people considering a TJR. The impairments might include reduced joint movement, pain on movement and muscle weakness; the resulting activities limitations might be difficulty climbing stairs, and/or difficulty getting onto a bus. Consequent participation restrictions might be inability to get to the shops, or to go to stay with grandchildren because of the stairs at their home. Research has shown that the relationship between the I, A, and P domains of the ICF are not simple, with other factors such as self-efficacy and co-morbidities acting as independent determinants of the relationships between these variables [17].

Commonly used methods to assess function do not link up well with the ICF [15], but they too can be split into a number of different categories. In general we can use one of three different approaches:

1) Self-assessment, in which the person tells us what they can and cannot do, usually via a questionnaire.

2) Functional tests carried out in a clinic or laboratory, which may be observed or measured in an objective manner, such as timing or motion analysis.

3) Real-life observation or assessment, which can include covert monitoring of the sort used in insurance case assessment, or the utilisation of modern accelerometers which can monitor what activities are undertaken over a prolonged period of time. (This approach may well take over in the future, but it will not be considered further as we have not been able to investigate it)

There are many different techniques that can be used to assess function within each of these three general categories, and several of the most popular ones mix up both the different categories within the ICF classification, and the different assessment approaches. Therefore it is perhaps not surprising that a recent systematic review found extensive variation in the outcome measures used in clinical trials of TJR [18], and that other studies have found low-modest correlations between the different methods [19-22].

It has been recommended that a combination of outcome measures should be used to assess function after TJR [19,23]. However, there are many reasons not to use a wide number of measures with every patient, whether in clinical practice or research. These include patient fatigue and burden, time constraints of clinic and research appointments and time taken to process and analyse multiple information sources. Therefore, there is a need for guidance about which outcome measures are the most useful in assessing function before and after TJR. The aims of this study are to compare the properties and responsiveness (ability to identify responders and non-responders to TJR) of a selection of commonly used measures that are either self-assessment tools or functional tests, to examine how well they relate to the ICF concepts of I, A and P, and to explore the changes in the measures and domains of outcome over time.

## Methods

The ADAPT study (Assessing Disability After Partial and Total joint replacement) is a single-centre cohort study currently underway at the Avon Orthopaedic Centre (AOC). The AOC, based in the Southwest of England, is one of the largest elective orthopaedic units in the UK, with approximately 800 hip operations and 800 knee operations performed in 2011[24]. The ADAPT study has been approved by Southwest 4 Research Ethics Committee (09/H0102/72) and all participants provide their informed, written consent to take part. The study is registered on the NIHR Clinical Research Network Portfolio (UKCRN ID 8311).

### Study duration

Recruitment into the study began in February 2010 and finished in November 2011. The 1-year follow-up for all participants is anticipated to be complete by December 2012.

### Inclusion criteria

Patients listed for one of the following operations were eligible: primary total knee replacement, revision total knee replacement, unicompartmental knee replacement, patellofemoral replacement, primary total hip replacement, revision total hip replacement or hip resurfacing. Patients were recruited from a diversity of surgical procedures so that outcome measures could be assessed across their full range.

### Exclusion criteria

Patients were excluded from the study if they lacked the capacity to provide informed consent. This was assessed by the research nurse in accordance with guidance from the integrated research application system, which is responsible for providing ethical approval in the UK, and the Mental Capacity Act of 2005 [25]. This decision was made by a research nurse if the patient met one of the following criteria: 1.) they could not understand the information relevant to the decision to participate 2.) they were unable to retain the information about the study 3.) they were unable to use or weigh that information as part of the decision-making process 4.) they were unable to communicate their decision about participation (whether by talking, using sign language or any other means). Another exclusion criterion was severe functional limitations such that the participant was unable to walk as this would prevent the participant from being able to attempt any of the functional tests which would make it impossible to collect essential data. This was assessed in the discussion between the research nurse and the potential participant about what participation would involve and was always a mutual decision by the researcher and the patient. Being unable to complete questionnaires in the English language was also an exclusion criterion because not all the validated questionnaires have been translated into other languages.

### Participant recruitment

Potential participants were identified from the arthroplasty waiting list by the code for the intended operation and sent a postal invitation. Patients who returned a reply form were telephoned by a research nurse to discuss participation. This included a full explanation of study involvement, and a preliminary eligibility assessment by asking about the intended operation and assessing understanding of the information provided. Those that did not reply or who were missed from the initial postal invitation list due to late scheduling of hospital appointments were approached by a research nurse when they attended the pre-operative assessment clinic. These patients were identified by daily checking of the clinic lists. If they were interested, eligibility was assessed and a full explanation of study involvement was provided. The first appointment was arranged then or they were telephoned a few days later if they needed time to consider. In order to explore whether the patients enrolled in the study were representative of those undergoing TJR, all eligible participants were entered on a database on which data about age, gender and postcode were recorded.

### Assessment times

Participants attend a mutually convenient research appointment, lasting approximately one hour, at the AOC. Appointments are scheduled before surgery and then at 3-months and 1-year after surgery. Assessments are conducted at 3-months post-operative to coincide with the standard clinical review, by which time most patients have experienced a large improvement in pain and function [26]. Assessments are also conducted at 1-year post-operative as outcomes can continue to improve up until this time point [26]. The inclusion of two post-operative assessments also allows exploration of outcome trajectories and comparison of rates of improvement between different outcome domains (e.g. pain, function, participation).

At the initial pre-operative appointment eligibility was confirmed, informed written consent was obtained and a questionnaire was given to participants to return by post before their operation. For the post-operative assessments, a questionnaire is sent out prior to the research appointment. At each time point, participants undergo a clinical assessment. These assessments are conducted by trained research nurses who follow standard operating procedures to ensure consistency and standardisation in data collection and who were assessed for competency in the examination procedures by a senior research nurse and orthopaedic surgeon. Risk assessments of the functional tests were undertaken and safe operating procedures specified. The data collected during the assessments are recorded by the research nurses on standardised proformas.

### Selection of outcome measures

Table 1 provides a summary of the functional assessment measures used in this study. The Table also provides an overview of the ICF domains included within each functional assessment measure, with classification of self-completed PROMs and clinician-administered measures based on the results of an expert consensus study by Pollard and colleagues [15]. There are a number of other measures that could have also been included in this study such as the Oxford hip and knee scores [27,28], Hospital for Special Surgery Score [29], Knee injury and Osteoarthritis Outcome Score [30] and Nottingham Health Profile [31]. However, to avoid participation burden and fatigue only a selection of measures was chosen, which are outlined below.

**Table 1:** Summary of measures used to assess function pre-operatively and at 3-months and 12-months post-operatively

	**ICF domains assessed**			**Mode of completion**	**Scoring**
	**I**	**A**	**P**		
Patient-reported outcome measures*					
WOMAC function subscale		++	+/−	Patient	0-68
Ab-IAP	++	++	++	Patient	I scale = 9–45, A scale= 17–85, P scale = 9-45
SF-12 Physical Component Score	+/−	++	+/−	Patient	0-100
MYMOP2^†^	+/−	+/−	+/−	Patient with assistance from research nurse	0-6
**Clinician-administered measures**^**#**^					
American Knee Society Score	+	++	-	Research nurse and patient	0-100
Harris Hip Score	+	++	+/−	Research nurse and patient	0-100
**Performance tests and motion analysis**^**#**^					
Timed 20-metre walk	-	++	-	Research nurse and patient	Time, difficulty, motion parameters
Timed Get up and go test	-	++	-	Research nurse and patient	Time, difficulty, motion parameters
Sit to stand to sit	-	++	-	Research nurse and patient	Completion, difficulty, motion parameters
Step tests	-	++	-	Research nurse and patient	Completion, difficulty, motion parameters
Single stance balance tests	-	++	-	Research nurse and patient	Completion, difficulty, motion parameters

ICF= International Classification of Functioning, Disability and Health, I=Impairments, A=Activity limitations, P=Participation restrictions.

The extent to which I, A and P is assessed within each tool is indicated using the following symbol system: - no items assessing domain, −/+ a small number of items assessing domain, + some items assessing domain, ++ the majority of items assessing domain.

*Scores are self-completed by the patient in a postal questionnaire, excepting MYMOP2 which is completed during a research appointment.

^†^The MYMOP2 asks patients to generate two symptoms (impairments or activity limitations) and one activity (which could be activity limitation or participation restrictions) that is restricted by their symptoms.

^**#**^Scores and tests are assessed by a research nurse during a research appointment. The patients are also invited to rate their performance on the functional tests.

### Patient-reported outcome measures

The following validated measures are used to provide disease-specific and generic self-reported measures of outcome:

***Western Ontario and McMaster Universities Osteoarthritis index (WOMAC) function scale***[32]: This disease-specific subscale, validated in OA patients, consists of 17 questions assessing the extent of function limitations when performing a range of daily activities. Responses are provided on a 5-point Likert-type scale.

***Aberdeen Impairment, Activity Limitation and Participation Restriction Measure (Ab-IAP)***[33]: This 35-item disease-specific measure, validated in OA patients, uses the ICF framework to assess disability and produces scores for I, A and P. Responses are provided on a 5-point Likert-type scale.

***Short-form 12 (SF-12)***[34]: This 12-item general health measure produces a Physical Component score and Mental Component score scale. Responses are provided as binary options (yes/no) or on a Likert-type scale.

***Measure Yourself Medical Outcome Profile 2 (MYMOP2) ***[35]: This patient-generated instrument allows participants to generate and rate the severity of two symptoms that are concerning them and one activity important to them that is restricted by the symptoms. Participants also rate their general well-being, duration of symptom 1 and medication usage for symptom 1. At follow-up, participants are asked to rate the severity of the symptoms and degree of restriction of the activity that they identified at the first data collection point. Ratings are provided on scales of 0–6. The MYMOP2 is completed during research appointments by participants with the assistance of research nurses.

Participants also complete a number of other questionnaires to assess factors that have been found to influence outcomes after TJR (Table 2). At each assessment time, participants complete the Hospital Anxiety and Depression Scale [36] and the WOMAC Pain and Stiffness Subscales [32]. Participants are also asked to rate how disabled they perceive themselves because of their joint problems and why, and to list three things that they are hoping for from their TJR. Pre-operatively, medical co-morbidities are recorded using the Functional Co-morbidity Index [37] and information is collected about socioeconomic status (marital status, living arrangements, educational attainment, employment status), joints affected by arthritis, and previous surgery on other joints. In the 1-year post-operative questionnaire, satisfaction with the outcome of surgery is assessed using the Self-Administered Patient Satisfaction Scale for Primary Hip and Knee Arthroplasty [38].

**Table 2:** Summary of measures used to assess factors influencing function

	**Dimensions**	**Mode of completion**	**Scoring**	**Assessment times**		
				**Pre-op**	**3-month**	**1-year**
WOMAC pain subscale	Joint pain	Patient	0-20	✓	✓	✓
WOMAC stiffness subscale	Joint stiffness	Patient	0-8	✓	✓	✓
Hospital and Anxiety Depression Scale	Depression, anxiety	Patient	0-21	✓	✓	✓
SF-12 Mental Component Score	Mental health	Patient	0-100	✓	✓	✓
Perceived level of disability	Function	Patient	0-10	✓	✓	✓
3 things hoping for from surgery	Expectations	Patient	Categorical	✓	✓	✓
Functional Co-morbidities Index	Co-morbidities	Patient	0-18	✓		
Arthritis and surgery in other joints	Co-morbidities	Patient	By joint/count	✓		
Socioeconomic status	Socioeconomic	Patient	Categorical	✓		
Self-Administered Patient Satisfaction Scale	Satisfaction	Patient	0-100			✓

### Clinician-administered measures

The Harris Hip Score is completed with hip replacement patients and the American Knee Society Score is completed with knee replacement patients.

***Harris Hip Score (HHS) ***[9]**:** This assessment measure provides a total score of between 0–100 (worst to best) collected over 4 domains. Function, which includes limp, use of assistive devices, walking distance, managing stairs, using public transport, sitting comfortably and putting on shoes and socks, is weighted the most heavily and is assigned 47 points. Pain is assigned 44 points. The physical examination involves assessing deformity (4 points) and range of motion (5 points).

***American Knee Society Score (AKSS)***[10]: This assessment consists of a Knee Score and a Function Score, both with a total score ranging from 0–100 (worst to best). The Knee Score incorporates examiner’s rating of patients’ pain (50 points) and a clinical assessment of stability (25 points) and range of motion (25 points), with deductions for flexion contracture, extension lag and misalignment. The Function Score consists of questions about walking distance (50 points) and stair climbing ability (50 points), with deductions for the use of walking aids.

### Performance tests

Before performing each of these tasks, participants are asked if they think they will be able to perform the task and estimate how difficult they think the test will be to perform on a 0–10 scale (no difficulty at all to impossible). After they have completed the test, they are then asked to rate how difficult the task actually was to perform on the same 0–10 scale. The research nurse conducting the assessment also provides a rating of how difficult it appeared to be for the participant to perform the task. If participants are unwilling to attempt the test or the research nurse is unhappy to proceed because of safety concerns, the test is not performed. All tests are performed without the use of supportive aids except the timed 20 metre walk and are described in the order in which they are performed.

***Timed 20-metre walk***[39]: Participants are timed as they walk a 20-metre straight distance on level ground at their normal, comfortable speed. If the participant normally uses a walking aid they are asked to try without it but if they feel unable to do so, they complete the test using the walking aid. The recorded outcome is the time taken to complete the test.

***Timed get up and go test***[40]: Participants sit on a height adjustable chair such that a 90° angle is formed when the femur is horizontal and the tibia vertical with their feet shoulder width apart and their arms crossed against their chest. Participants are timed as they stand up from the chair without using their hands, walk at a normal pace past a marker three metres away, turn around, walk back and sit down again. The recorded outcome is whether participants are able to complete the activity and how long it took.

***Sit-to-stand-to-sit***[41]**:** Participants sit on a height adjustable chair as described for the previous test. Participants then stand up, wait two seconds and sit down again without using their hands. The recorded outcome is whether participants are able to complete the activity.

***Step test***[42]: Participants step up onto a 20cm high block leading with the contra-lateral leg, wait two seconds, and then step down from the block with the index leg leading, without using their arms. The test is then repeated with the index leg leading. If participants successfully completed this test, the test is repeated with a 30cm block. The recorded outcome is whether participants are able to complete the activity.

***Single stance balance test***[43]: Participants stand with their feet together facing the research nurse and place their palms gently on top of the research nurses’ palms. Participants then lift their index leg and attempt to balance on their contra-lateral leg for 15 seconds. If the participant loses balance within three seconds, then the test is reattempted. If the participant loses balance before 15 seconds, the length of time is recorded. This test is then repeated while balancing on the index leg. If these tests are completed successfully, the participant repeats the tests with no stability support from the research nurse. The recorded outcome is whether participants are able to maintain the stance for 15 seconds.

### Inertial sensor based motion and gait analyses

Movement analysis by body-fixed inertial sensors containing accelerometers and gyroscopes enables the objective assessment of the translational and angular movements of body segments outside a gait laboratory [44,45]. We used a single 3D inertial sensor (41x63x24mm; 39g; Microstrain Inertia Link) containing accelerometers (±5g) and gyroscopes (±300°/s) along the three orthogonal axes in frontal, sagittal and transverse plane and positioned centrally between both posterior superior iliac spines to measure trunk movements near the center of gravity. Based on the 3D linear accelerations, angular rates and angular positions put out by the sensor and send wirelessly to a PC at a 100Hz sampling frequency via a Bluetooth link, analysis algorithms calculate motion parameters such as step frequency, step asymmetry or trunk sway.

The inertial sensor was used to derive motion parameters from a battery of movement tasks which are clinically feasible to perform during a routine outpatient visit and which challenge the patient’s functional capacity in different ways: 1) locomotion (walking); 2) transfers (sit-stand-sit, get up and go); 3) rising and descending (step test) and 4) balance tests (single leg stance). The walk test [46] and the step-test are repeated twice and the sit-stand-sit test is repeated three times to derive representative mean values or study possible effects of fatigue or warming up.

### Data collection

Information on body mass index, diagnosis, side of surgery, type of surgery and surgical approach is extracted from participants’ medical records.

### Data recording

All data is entered into a password-protected database by research nurses or study administrators. The study is overseen by an independent Steering Committee which meets every 6 months to discuss the progress of the study. Data monitoring, which involves double data entry and quality checks, is conducted every 3-months. All data will be cleaned before data analysis is performed. Any inconsistencies will be collegially discussed by an internal board of researchers involved in the data collection.

### Sample size

This study will involve exploratory analysis to compare different measures to assess function after TJR. Therefore, no formal sample size calculation was performed, although we aimed to recruit a sufficient number of patients to allow meaningful data analysis. We approached all patients listed for surgery with participating surgeons between February 2010 and November 2011. Previous longitudinal studies comparing measures of function in an orthopaedic population have included between 30 and 200 patients [22,47-54]. Recruitment for this study is complete and 133 knee replacement patients and 130 hip replacement patients have been recruited, making this one of the largest longitudinal studies to compare measures of function in TJR patients. The 133 patients listed for a knee surgery are planned to receive a primary total knee replacement (n=51), a revision total knee replacement (n=45), a unicompartmental knee replacement (n=32) or a patello-femoral replacement (n=5). The patients listed for a hip surgery are planned to receive a primary total hip replacement (n=78), revision total hip replacement (n=44) or a hip resurfacing (n=8).

### Data analysis

This section is an overall presentation of the quantitative and qualitative analysis plan and does not intend to provide a detailed account of all forthcoming analysis. Nested analyses using sub-groups of patients are also planned.

#### Quantitative analysis

Participation rates will be compared by age, gender and postcode with Poisson modified regression[55]. Descriptive statistics alongside measures of precision (95% Confidence Intervals) will be used to present the measures at each assessment time. The first investigations will focus on the pre-operative period. Receiver Operating Characteristic curve, correlation analyses or generalized linear models (GLM)[56] will be considered to study the relations between measures aiming to describe the same domain (I, A or P) or between performance test, self-assessed and clinically-administrated measures. We will also study how the various measures of A correlate to P (measured with the Ab-IAP).

For each self-reported and clinician-administered assessment of function, internal responsiveness will be investigated by dichotomising patients into those that do and do not improve as a result of surgery using established methods such as the “OMERACT-OARSI responder criteria” or the “Minimum Important Difference” [57,58]. External responsiveness will be explored by calculating the minimum clinically important difference (MCID) for each measure, using the Self-Administered Patient Satisfaction Scale as the clinical anchor to derive MCIDs.

The role of patients’ pre-operative characteristics (Table 2) on responsiveness will be studied with GLMs. The distribution of the pre-operative measures (skewness, peakedness and percentiles) will be used to derive patient classifications by levels of I, A or P. Patient profile will also be adjusted for in the multivariate analyses. Concordance in responsiveness between measures will be studied. These comparisons will be re-conducted within each pre-operative I,A or P patient profiles. Adjusted and non adjusted GLMs will be used to study the association with responsiveness and the outcomes of performance tests for measures specific to the A domain.

The longitudinal aspect of the data collection will allow the study of the change in the measures between the pre-operative period and two time points after surgery. Generalized linear mixed or marginal models[59] will be used to study the patients’ trajectories and investigate the impact of pre-operative and time-dependant patients characteristics. Particular attention will be paid to the distributions of the raw scores of each measure and appropriate transformation or categorisation will be conducted. Tests such as the Log-likelihood ratio test or the Wald test will be used to identify statistically significant effects or interactions (*P*<0.05). Missingness will be investigated and if necessary Multiple Imputation by Chained approach will be considered and estimates combined with Rubin’s rules. The analyses will be conducted with Stata 11.2.

#### Inertial sensor analysis

Real-time data from the inertial sensor will be stored on a computer with a sampling frequency of 100Hz. After manually selecting the start and end of each test, self developed algorithms based on previously published principles [60] will automatically derive a motion parameter set for each test e.g. for gait, spatiotemporal parameters will be derived after heel strike detection based on the zero crossing method described by González and colleagues [61] and pelvic angles will be derived by common peak detection methods. Automated peak detection after signal filtering is also the main principle to derive the motion parameters for the step, get up and go, sit-stand-sit and the balance tests. In addition, measuring the time to task achievement for each test is more accurate and reproducible from the sensor signal than from a stop watch. For tests with repetitions (e.g. sit-stand-sit) the average values will be reported. All signal analysis and motion parameter calculations will be programmed in MATLAB® (MathWorks®) version R2009a.

#### MYMOP2 analysis

To characterise the symptoms that matter most to patients, symptoms identified by participants completing the MYMOP2 will be inductively categorised into groups. Mean profile scores will be calculated from patients’ rating of their symptoms. Taken together, these will provide information about the issues that patients think most important and extent to which profile scores in different categories have changed between pre-operative and post-operative time points.

## Discussion

The existence and application of a range of different measures to assess function before and after TJR presents a number of challenges. Without consistency across studies, it is hard to compare results or conduct meta-analysis. In the absence of information about how measures compare, choice of outcome measure may be overly influenced by practical concerns or habit. This study aims to address some of these issues by comparing the properties and responsiveness of a selection of commonly used measures for assessing function before and after TJR, examining how well they relate to the ICF concepts, and exploring the changes in the different measures and domains of outcome over time. By including a number of outcome measures, the longitudinal study design enables an examination of patients’ trajectories after TJR taking into account physical, psychological and social participation trajectories.

The study has some limitations that should be acknowledged. The order of the performance tests is not randomised and therefore participant fatigue may influence the results. The assessments are all conducted at a hospital, rather than as home visits, to ensure that the tests are performed in a standardised environment and sufficient space is available for the walking test. However, this could influence the characteristics of the participants because it is possible that individuals with the greatest functional limitations will be less likely to be able to attend the hospital for the research appointments. The strengths of the study include the relatively large sample size for this type of study, its longitudinal design and the standardisation of assessment methods through training and use of standard operating procedures. Data collection for this study will be complete by December 2012 and results from the study will be analysed during the course of 2013.

## Abbreviations

TJR: Total joint replacement; ICF: International Classification of Functioning, Disability and Health; I: Impairment; A: Activity limitations; P: Participation restrictions; PROMs: Patient-reported outcome measures; OA: Osteoarthritis; ADAPT study: Assessing Disability After Partial and Total joint replacement; AOC: Avon Orthopaedic Centre; WOMAC: Western Ontario and McMaster Universities Osteoarthritis index; Ab-IAP: Aberdeen Impairment, Activity Limitation and Participation Restriction Measure; SF-12: Short form-12; MYMOP2: Measure Yourself Medical Outcome Profile 2; HHS: Harris Hip Score; AKSS: American Knee Society Score; GLM: Generalized linear models; MCID: Minimum clinically important difference.

## Competing interests

The authors declare that they have no competing interests.

## Authors’ contributions

All authors conceived and designed the study. All authors drafted the manuscript, revised it critically for important intellectual content and have given final approval of the version to be published. All authors read and approved the final manuscript.

## Pre-publication history

The pre-publication history for this paper can be accessed here:

http://www.biomedcentral.com/1471-2474/13/220/prepub
